# Efficient mass transport by optical advection

**DOI:** 10.1038/srep14861

**Published:** 2015-10-06

**Authors:** Veerachart Kajorndejnukul, Sergey Sukhov, Aristide Dogariu

**Affiliations:** 1 CREOL, The College of Optics and Photonics University of Central Florida, 4000 Central Florida Boulevard Orlando, Florida 32816, USA

## Abstract

Advection is critical for efficient mass transport. For instance, bare diffusion cannot explain the spatial and temporal scales of some of the cellular processes. The regulation of intracellular functions is strongly influenced by the transport of mass at low Reynolds numbers where viscous drag dominates inertia. Mimicking the efficacy and specificity of the cellular machinery has been a long time pursuit and, due to inherent flexibility, optical manipulation is of particular interest. However, optical forces are relatively small and cannot significantly modify diffusion properties. Here we show that the effectiveness of microparticle transport can be dramatically enhanced by recycling the optical energy through an effective optical advection process. We demonstrate theoretically and experimentally that this new advection mechanism permits an efficient control of collective and directional mass transport in colloidal systems. The cooperative long-range interaction between large numbers of particles can be optically manipulated to create complex flow patterns, enabling efficient and tunable transport in microfluidic lab-on-chip platforms.

The transport of mass is fundamental for a broad spectrum of physical processes occurring naturally or in externally controlled circumstances. In biology, the intracellular and intercellular transport occurs within fluids at low Reynolds numbers with viscous forces dominating inertia. In this regime, besides diffusion, advection processes control the transport over larger length scales^1^,^2^,^3^. For instance, in large plant cells, the cytoplasmic streaming driven by the movement of myosin motors along actin filaments is responsible for circulating microscopic particles or organelles^2^,^3^. This efficient mass transference assisted by the fluid flow happens at large Péclet number (Pe) where advection dominates diffusion. Another function of high Pe fluid flow is enhancing the metabolites mobilization through the rotational streaming of vacuolar fluid^4^.

A number of biomimetic approaches are being pursued to mimic or even surpass nature’s performance. Among these, the transport of small entities of matter, ranging from individual molecules to micron size particles, is of considerable interest. At room temperature, due to the small sizes involved, manipulation and transport at high or adjustable Pe number is a challenging task.

The action of optical fields provides unique opportunities for creating and controlling liquid flows and for manipulating particles at small scales. Optical fields induce mechanical effects through energy and momentum transfer. It is known that illumination of a homogeneous light-absorbing liquid induces heating that may give rise to convection. Absorption can also produce thermocapillary convection flows^5^. Droplet coalescence can assist fluid flows by making use of photothermal nanoparticles^6^. Fast micro-particles motion (up to 10^4^ m/s) can be achieved by a shock acceleration method based on explosive evaporation of a nanoparticle’s surface as a result of high-intensity laser irradiation^7^. Even though thermally driven forces can be substantial, the electromagnetic to thermal energy conversion is not always desired. Sometimes, the use of high-level optical irradiance may not be possible and, moreover, the unavoidable thermal modifications of the environment also influence the diffusive transport, which may cause problems when attempting to activate mass transport at large Péclet numbers.

The other option to generate mechanical action is by direct momentum transfer, which does not influence the thermal properties of the environment^8^,^9^. Unfortunately, the efficiency of this process is usually rather small and the approach may not be sufficiently effective unless the optical irradiance is increased up to unacceptable levels. A number of papers are devoted to the study of propulsion of micro-particles along plasmonic surfaces^10^,^11^ or waveguide structures^12^,^13^ employing evanescent waves. The reported velocities for 10 to 20 μm diameter polystyrene spheres contained in water and placed onto a gold interface are 4…1.5 μm/s under illumination intensity 33 μW/μm^2^ 11. With similar irradiation intensities 38 μW/μm^2^, 1…27 μm diameter polystyrene spheres were moved with an average speed of 8 μm/s on top a glass prism illuminated in total internal reflection^12^. Much higher propulsion velocities of 450 μm/s for 15–20 μm polystyrene microspheres in water were demonstrated in ref. 14 for guided powers as low as 43 mW. However, this power was squeezed into a microfiber with 1.5 μm diameter that makes effective intensity (~50 mW/μm^2^) many orders of magnitude higher than that in above mentioned experiments.

Here we will show that the transport of particles can be significantly enhanced through an efficient use of the momentum imparted to surrounding colloidal medium. In this Letter we prove that high Pe number regimes can be achieved by taking advantage of optically induced advective flows in a colloidal system. Most importantly, we demonstrate both theoretically and experimentally that these large Pe numbers can be reached at low levels of optical irradiance. In particular, we will show that the transport velocities of the same magnitude as in refs 11,12 can be achieved with two orders of magnitude less intensity.

## Theoretical background

Let us consider an unfocused beam of light propagating through a dilute colloidal suspension. Disregarding gradient forces, the optical scattering force acting on colloidal particles can be described as


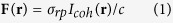


where 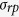 is the radiation pressure cross-section and 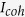 is the *coherent* part of propagating beam. We note that only this coherent part produces an ordered movement of particles while the scattered or *incoherent* part results in randomly oriented optical forces. These random forces can lead to other interesting effects^15^, but they are usually much weaker and, therefore, not relevant for the case discussed here. The decay of the coherent part of the beam in scattering medium is defined by the scattering length^16^ 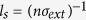, where *n* is the number density of particles and 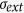 is the associated extinction cross-section. At high intensities, a nonlinear dynamics may also develop due to an interplay between optical forces and the modified local particles concentration^17^,^18^. These extreme conditions however require irradiances many orders of magnitude higher than what is considered here.

Subjected to the field of forces **F**(**r**) in Eq. (1), the velocity of particle *i* can be described within the additive pair-interaction approximation as a sum over stokeslets^19^


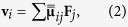


where 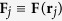 is the force acting on particle *j*. The summation in Eq. (2) is performed over all the particles affected by nonzero forces. The hydrodynamic interaction between the point-like particles *i* and *j* of a dilute colloidal system is described by the Oseen tensor^19^ 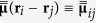



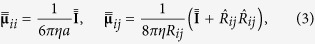


where *a* is the particle radius (the particles are assumed to be monodispersed), *η* is the dynamic viscosity of the surrounding liquid, 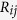 is the distance between centers of the *i*-th and the *j*-th particles, 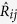 is the unit vector in a direction from particle *j* to particle *i*, and 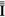 is a unitary matrix. One can see from Eq. (3) that the mobility matrix 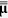 is inversely proportional to the interparticle distance 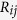. This slow dependence on 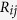 makes the sum in Eq. (2) be strongly influenced by the magnitude and the spatial characteristics of the force field. In particular, it follows that by orchestrating the particle motion with a directionally uniform field of forces, one can significantly increase particles’ velocities because in Eq. (2) all the terms enter the sum with the same sign.

In some cases, the distance between colloidal particles is much smaller than characteristic dimensions of the beam (the width and the scattering length 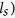 and the summation in Eq. (2) can be replaced by integration. In this case, instead of forces acting on discrete colloidal particles, one can consider volume forces acting on the fluid itself. This continuum description has been used previously in a number of situations^20^,^21^,^22^. When this formulation is applied to absorbing liquids in finite size vessels, the speed of optically-induced convective flows can be approximated as^20^


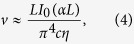


where 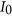 is the intensity, *α* can be set to be equal to 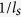, *L* is the depth of the container, *c* is the speed of light.

Starting from the elementary description of the velocities in Eq. (2), we derived an expression for the velocity of “optical streaming” in the case of deep containers 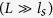 and wide illumination beams (see Supplementary materials for details):


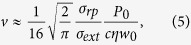


where 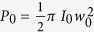 is the total power carried by the beam with a waist size 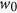. As one can see, the expression is qualitatively similar to Eq. (4), although for deep containers the velocity of optically induced flow is determined mostly by the parameters of the beam rather than the properties of the colloidal particles. An expression for the velocity of optically induced flow in a general case can be found in Supplementary materials. One can easily estimate that, for nanometer size particles and optical beam diameters ranging from microns to millimeters, the flow velocity can be three to six orders of magnitude higher than the speed of an individual colloidal particle. The colloidal particles moving together drag the surrounding liquid and create a significant advective flow. Target particles placed inside this colloid can be caught in this advective flow and can acquire velocities much higher than usually experienced when placed in transparent fluids.

When estimating the optical streaming velocity in Eq. (5), we replaced the discrete summation in Eq. (2) with an integration in a manner similar to the hydrodynamic description of refs 20, 21, 22. However, there could be situations where the direct summation is more appropriate. This is the case, for instance, of finite-size, heterogeneous colloidal systems consisting of particles with different properties and also the case of colloids in highly focused and structured fields. These circumstances make the summation description in Eq. (2) ideal for describing micro-fluidic applications. It could be noted that the discrete-colloid approach used in Eq. (2) can be seen as an analog of the discrete dipole approximation (DDA) in electrodynamics^23^ and, correspondingly, the area of application of discrete-colloid approach can be similar to the one of DDA.

### Experimental verification of the concept of advective transport

**Figure 1:**
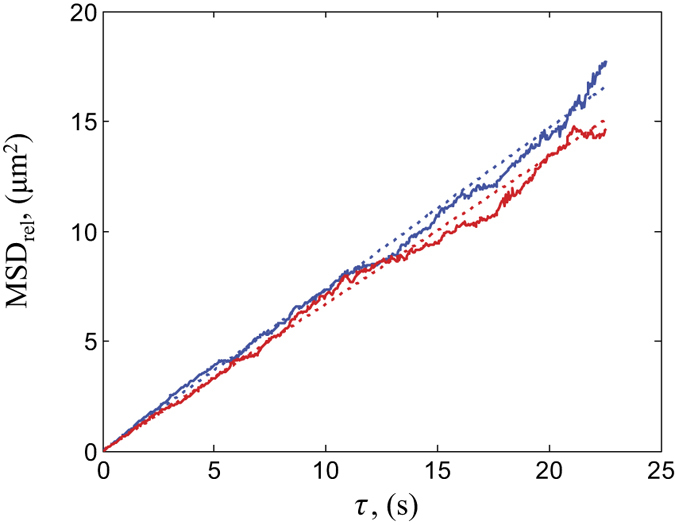
The temporal evolution of the relative mean square displacements of diffusing target particles. Particles are placed on the surface of pure water (red color) and on the surface of a colloidal suspension of polystyrene nano-particles (blue color).

To verify the proposed concept of particle transport, we performed systematic experiments with microscopic 4.5 μm-diameter polystyrene (PS) particles. First, we investigated transport properties in the absence of optical advection. PS particles were dispersed onto the surface of water over an area of ten millimeters in diameter. The method for depositing microspheres on the liquid surface was described in ref. 24 (see Methods for a detailed description). The motion of the target particles at the air-water interface was imaged through a 10 × microscope objective (NA 0.25, Olympus) and recorded by a CCD camera (Andor sCMOS) with a frame rate of 25 f.p.s. The motion of PS particles was recorded and particle-tracking techniques were applied to analyze the particles’ trajectories as described in Methods. In the absence of the light illumination, the PS particles experience a Brownian motion. The diffusion coefficient can be experimentally measured from the relative mean square displacements 
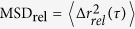
 as described in Materials and Methods section^25^. Figure 1 shows the relative mean square displacements of PS particles as a function of time lag *τ*. The corresponding diffusion coefficient is 0.0836 ± 0.0002 μm^2^/s, which corresponds to an average displacement of 2.68 μm over 10 s duration. Experimentally found diffusion coefficients are approximately two times lower than the theoretically estimated value (~0.22 μm^2^/s). The theoretical value of diffusion coefficient for surface bound particles is found by knowing the contact angle (that is ≈90° for PS and water^26^) and corresponding submergence depth for polystyrene^27^. In particular, PS particles are half-submerged into water that makes their diffusion coefficient twice larger than that for the particles in a bulk. The reduction of the diffusion coefficients of the target particles can be due to surface contamination.

**Figure 2:**
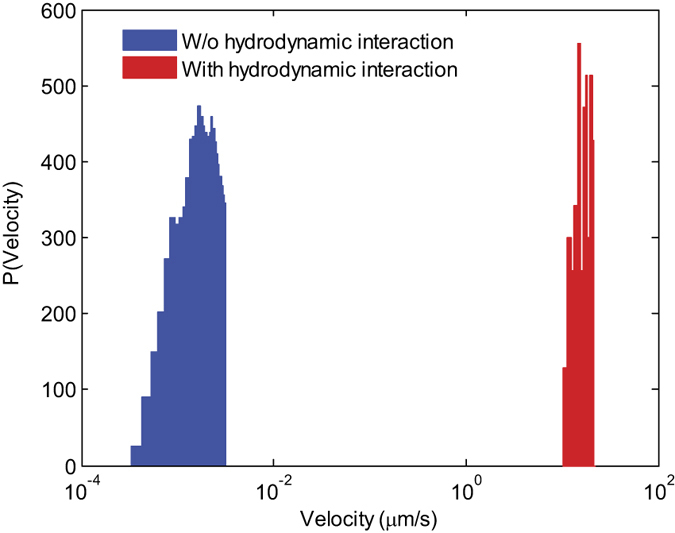
Probability density functions of velocities for 4.5 μm PS target particles propelled by laser irradiation. Target particles are uniformly distributed on the surface of pure water (Blue color) and on the surface of a monodisperse colloidal suspension of polystyrene nano-spheres (Red color) under the laser illumination with optical powers of 2.5 and 0.3 W, respectively.

After measuring the diffusion coefficient, the system was illuminated by a linearly polarized monochromatic unfocused light from a CW laser (Coherent Genesis CX Series model; wavelength 532 nm; optical power 2.5 W; beam diameter 2.2 mm) at the incident angle of 27°. Two 532 nm notch filters (OD4, from Edmund) were used to remove the laser light scattered towards the CCD camera. At the operating wavelength of 532 nm, the absorption in polystyrene and water is minimal and causes only negligible thermal contributions meaning that all the mechanical effects of light can be attributed to the exchange of linear momentum. We found that the micro-particles placed on the surface of pure water are pushed along the beam propagation with a maximum velocity *v* ≈ 0.72 μm/s. The distribution of velocities is depicted in Fig. 2 with blue color. The Peclet number for the particles on a surface of pure water can be estimated to be 2*av*/*D* ≈ 39. This is relatively high, but we emphasize that this value was reached by using 2.5 W illumination power.

**Figure 3:**
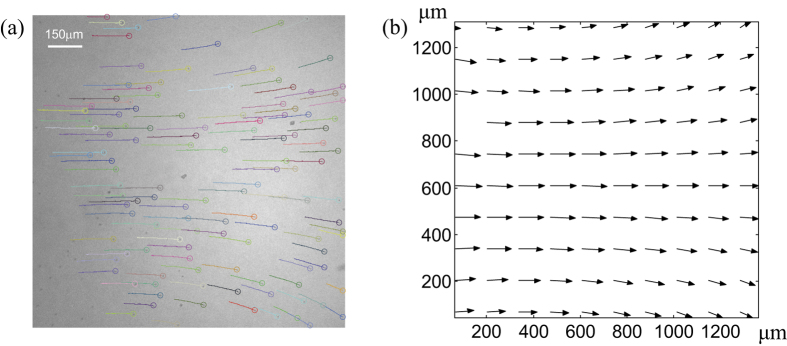
Advective transport of target particles. (**a**) Trajectories of target particles during the 10 s of laser illumination. The ends of trajectories are indicated by small circles. (**b**) Spatial distribution of time-averaged velocities. The length of the arrows is proportional to the speed while their orientations indicate the local direction of particles’ motion.

Next, we estimated the effectiveness of the micro-particles transport in the presence of advection. Polystyrene microspheres were placed onto the surface of colloidal suspensions containing 0.3%v/v colloidal concentration of 200-nm-diameter polystyrene particles. The measured diffusion coefficient in the absence of external illumination was 0.0919 ± 0.0002 μm^2^/s (see Fig. 1), which is close to the case of PS particles placed on the surface of pure water. Following the measurement of diffusion coefficient, the colloidal system was illuminated by a CW laser beam with 0.3 W optical power and with all other parameters being the same as in the previous case. After switching on the illumination, the target particles on a surface started immediate motion (see Supplementary Video 1) and continued to move until light was turned off. An example of reconstructed trajectories of floating manipulated particles is shown in Fig. 3a. The center of the illuminating beam lies in the middle of the image and, as can be clearly seen, the particles in that vicinity experience a directional motion along the beam propagation. The spatial distribution of velocities of transported particles is shown in Fig. 3b while the corresponding histogram of the velocity magnitudes is displayed in Fig. 2 (red color). The maximum velocity of the transported particles observed in experiment is 22.3 μm/s. It can be clearly seen that, in this case, the velocities are one order of magnitude larger than the velocities measured on the surface of pure water. The corresponding Peclet number is over 1000(!). It also should be reminded that this value was obtained for an optical power of illumination 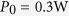, which is almost one order of magnitude lower than the power used in the case of pure water. Remarkably, in spite of lower illumination intensity, the corresponding Peclet number increased 30 times in comparison to the case of pure water interface! With a diameter of incident beam being 2.2 mm, the irradiation intensity in our experiment was 0.16 μW/μm^2^ that is 2 orders of magnitude less than in the experiments with evanescent waves^11^,^12^,^13^. With this low intensity level we could achieve several times higher propulsion velocities because of an efficient ‘recycling’ of the lost energy through the colloidal particles.

Additional results illustrating the optically stimulated advection in a different type of colloid are presented in Supplementary materials. That experiment made with silica colloidal particles also shows the significant increase of Peclet number.

In principle, the achievable advective transport velocities can be even higher. To prove that, we performed extended numerical calculations of advective flows with the same parameters as in experiment (see Supplementary materials) while considering the situation of negligible contributions from the container walls. Calculations show that the velocity of advective flows can achieve a maximum speed of ≈190 μm/s. The measured velocities in the experiment are an order of magnitude lower, which could be explained by the finite dimensions of the cuvette; it is known that backflow of displaced water outside the region of action of optical forces apparently slows down the induced advection^22^. In addition, the reduced measured velocities of the target particles could be a consequence of possible surface contamination that also explains lower measured diffusion coefficients of PS particles.

We note that the proposed concept of controlled transport can be generalized and implemented with different external driving forces. For instance, fluid flows can be generated by applying time-dependent biaxial magnetic fields to magnetic platelet suspensions^28^,^29^. Complex flow patterns can be externally controlled by the specific properties of both the applied fields and suspended particles, enabling tunable heat and mass transport^29^. However, the magnetic forces are usually much stronger than the optical ones leading to higher velocities of turbulent flows at high Re numbers that may alter the transport.

## Conclusions

In conclusion, we proposed and demonstrated ability of enhanced transport of microscale particles through advection mechanism that can be controlled optically. The electromagnetic fields can be easily structured to manipulate the complex flow pattern, enabling an efficient and tunable transport of mass.

The experiments in this paper were performed on micrometer-size particles such that their movement can be directly visualized. However, the concept of optically induced advective transport would be especially beneficial for nano-sized objects. In the case of nano-particles the radiation pressure cross-section is extremely small, which makes the direct optical manipulation almost impossible. In addition, the diffusion coefficient of nano-particles is very large making the directional manipulation even harder. Also, because of diffraction limit, the light cannot be deployed only over the targeted area of a particle and a large part of radiation is practically lost. For example, the Peclet number for an individual 200 nm particle used in our colloidal system would reach 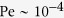 for a Watt-level illumination confirming that the radiation pressure by itself is completely ineffective for manipulating nanometer size particles. The proposed concept for advective transport allows us to ‘recycle’ the lost energy and to use it efficiently for manipulating the target particles. As such, the Péclet number of 200 nm particles caught in the advective flow would be of the order of unity even when the irradiation power is one order of magnitude smaller (~100 mW).

The new optofluidic technique permits to effectively regulate the mass transport in a remote, noninvasive, and, most importantly, in a power efficient manner, which should be of interest for microfluidic lab-on-chip platforms.

## Materials and Methods

### Sample preparation

The suspension of 4.5-μm-diameter PS particles with Coefficient of Variance (CV) 7% (Polysciences, Inc.) was dissolved into methanol (99.9 mol% pure, FisherScientific). Single small drop of the solution (~6 μl) was gently released from a 10 μl syringe (World Precision Instruments) onto the surface of pure water or colloidal suspension. Upon the release of the solution, methanol evaporated rapidly, spontaneously forming a well dispersed monolayer of micron sized particles on the interface as can be observed in Fig 3a. This prepared sample was contained in a cavity inside a large metallic plate. The container was covered by a glass coverslip to prevent excessive particle movement due to external air currents.

### Particle tracking

The image processing algorithm based on the determination of brightness-weighted centroids was implemented in a homemade MATLAB code to precisely locate the particle centers in each frame. Linking particles from different frames into trajectories was performed using the third party MATLAB code^30^.

### Determination of diffusion coefficient

In the absence of laser illumination, the target particles experience a Brownian motion. The diffusion coefficient *D* can be determined from a relative mean square displacement of pairs of particles: 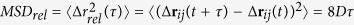, where 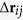 is the vector difference between positions of *i*-th and *j*-th particles^25^. Evaluating the relative components eliminates the influence of the synchronized motion of the particles generated by inevitable external current. In order to further eliminate the possible effects of nonuniformity of macroscopic flows, only particle pairs floating within ~90 μm of each other were considered.

## Additional Information

**How to cite this article**: Kajorndejnukul, V. *et al.* Efficient mass transport by optical advection. *Sci. Rep.* **5**, 14861; doi: 10.1038/srep14861 (2015).

## Supplementary Material

supplementary video 1

supplementary video 2

Supplementary Information
