# Supplementary material for: Efficient mass transport by optical advection
Source: Sci Rep. 2015 Oct 6;5:14861. doi: 10.1038/srep14861 (PMC4594360; doi:10.1038/srep14861)
Supplement: Supplementary Information [file srep14861-s3.pdf]

Supplementary information:

Efficient mass transport by optical advection

Veerachart Kajorndejnukul, Sergey Sukhov, and Aristide Dogariu

CREOL, The College of Optics and Photonics University of Central Florida, 4000 Central Florida Boulevard Orlando, Florida 32816, USA

1. Velocity of particles in a colloid illuminated by Gaussian beam

Here we analytically estimate the velocity of optically induced advective flows in colloidal system depending on parameters of illumination. Let us consider infinite liquid medium with semi-infinite space $z < 0$ containing colloidal particles. We assume that Gaussian beam is incident normally to the liquid-colloid interface. Each particle inside the colloid experience optical forces induced by the beam. We assume that the beam is not focused so that gradient forces would be negligible in comparison to scattering force. We also assume that the colloid is dilute so that there are no gradient forces because of fast decay of intensity into colloidal system[1]. In this approximation the force acts along the propagation direction of the beam, $-z$ direction. The scattering force is determined by the coherent part of intensity I_{coh} (see main text for explanations) and defined by the following expression[2]:

$$F(\mathbf{r}) = \sigma_{rp} I_{coh}(\mathbf{r})/c\,,\ \sigma_{rp} = \sigma_{ext} - \sigma_{sca}\langle\cos\theta\rangle\,, \tag{S1}$$

where σ_{rp}, σ_{ext}, σ_{sca} are the radiation pressure, extinction, and scattering cross-sections, respectively, $\langle\cos\theta\rangle$ is the scattering asymmetry parameter, c is the speed of light in the surrounding medium. Intensity of the coherent part of propagating beam $I_{coh}(\mathbf{r})$ is defined by the following formula[3]:

$$I_{coh}(z,\rho) = I_0 \exp(-2\rho^2/w_0^2)\exp(-2z/l_s)\,, \tag{S2}$$

where ρ is the radial coordinate, w_0 is the waist size of the Gaussian beam, $l_s = (n\sigma_{ext})^{-1}$ is the scattering length and n is the number density of colloidal particles, $1/n = 4\pi a^3/(3f)$, a and f are the radius and volume fraction of colloidal particles.

For a dilute colloid, the velocity of colloidal particle *i* in the location defined by radius-vector $\mathbf{r}_i$ can be found in an approximation of hydrodynamic pair interaction of point-like particles[4]:

$$\mathbf{v}(\mathbf{r}_i) = \sum_j \overline{\overline{\boldsymbol{\mu}}}(\mathbf{r}_i,\mathbf{r}_j)\mathbf{F}(\mathbf{r}_j)\,. \tag{S3}$$

$\overline{\overline{\boldsymbol{\mu}}}(\mathbf{r}_i,\mathbf{r}_j)$ is the Oseen tensor[4] $\overline{\overline{\boldsymbol{\mu}}}(\mathbf{r}_i,\mathbf{r}_j) = (\mathbf{I} + \hat{R}_{ij}\otimes\hat{R}_{ij})/(8\pi\eta R_{ij})$ and $\overline{\overline{\boldsymbol{\mu}}}(\mathbf{r}_i,\mathbf{r}_i) = \mathbf{I}/(6\pi\eta a)$. Here $R_{ij} = |\mathbf{r}_i - \mathbf{r}_j|$ is the distance between centers of the *i*-th and the *j*-th particles, $\hat{R}_{ij} = (\mathbf{r}_i - \mathbf{r}_j)/R_{ij}$ is a unit vector in a direction from particle *j* to particle *i*, and $\overline{\overline{\mathbf{I}}}$ is unitary matrix, symbol $\otimes$ denotes outer product, η is the dynamic viscosity of surrounding liquid.

To obtain analytical expressions, we consider particles located at the axis of Gaussian beam. From symmetry considerations, only *z*-component of the velocity exits for these particles. Taking into account that

force $\mathbf{F}$ also has only z-component, only μ_{zz} component of the matrix $\overline{\overline{\boldsymbol{\mu}}}(\mathbf{r},\mathbf{r}_j)$ survives in expression (S3). In a typical situation, the distance between colloidal particles is much smaller than characteristic dimensions of the beam (w_0 , l_s) and summation in Eq.(S3) can be replaced by integration. In an initial instant of time when the light is just turned on and plain liquid-colloid interface is still unperturbed, the velocity of colloidal particles near the beam center can be evaluated from the following expression:

$$v_z(z,\rho=0)=\frac{F_z(z)}{6\pi\eta a}+\frac{1}{8\pi\eta\sigma}\int_{V-\sigma}\mu_{zz}(\mathbf{r},\mathbf{r}')F_z(\mathbf{r}')d\mathbf{r}' . \quad \text{(S4)}$$

The integration in Eq.(S4) is performed over the whole volume of colloid excluding volume $\sigma = 1/n$ around the particle at observation point z. For observation points close to the surface ($z \to 0$), the integral over semi-infinite volume V in Eq.(S4) can be calculated analytically (evaluation of integral was performed in Wolfram Mathematica 9.0):

$$\frac{1}{8\pi\eta\sigma}\int_{V}\mu_{zz}(\mathbf{r},\mathbf{r}')F_z(\mathbf{r}')d\mathbf{r}'=\frac{P_0\sigma_{rp}}{8\pi c\eta\sigma}\Phi\left(\frac{w_0}{\sqrt{2}l_s}\right), \quad \text{(S5)}$$

where $P_0 = \frac{1}{2}\pi I_0 w_0^2$ is the total power of Gaussian beam, $\Phi(x)=(\sqrt{\pi}x-1)-\exp(-x^2)(1-x^2)(\mathrm{Chi}(x^2)-\pi\,\mathrm{erfi}(x)+\mathrm{Shi}(x^2))$, $\mathrm{Chi}(z)$ is the hyperbolic cosine integral function

$$\mathrm{Chi}(z)=\gamma+\ln z+\int_0^z\frac{\cosh t-1}{t}dt$$

with $\gamma = 0.577...$ being Euler's constant, $\mathrm{Shi}(z)=\int_0^z \sinh(t)/t\,dt$ is the hyperbolic sine integral function, $\mathrm{erfi}(z)=\mathrm{erf}(iz)/i$ is the imaginary error function .

The integral over exclusion volume σ can be estimated under assumption that exclusion volume is much smaller than characteristic scale of change of optical forces ($a/f^{1/3} << w_0, l_s$):

$$\frac{1}{8\pi\eta\sigma}\int_{\sigma}\mu_{zz}(\mathbf{r},\mathbf{r}')F_z(\mathbf{r}')d\mathbf{r}'=F_z(\mathbf{r})\frac{2f^{1/3}}{\eta a} . \quad \text{(S6)}$$

After substitution of Eqs.(S5), (S6) into (S4) one gets the final expression for the velocity of colloidal particles near the interface:

$$v_z(\mathbf{r}\to 0)=\frac{F_z(0)}{\eta a}\left(\frac{1}{6\pi}-2f^{1/3}\right)+\frac{P_0\sigma_{rp}}{8\pi c\eta\sigma}\Phi\left(\frac{w_0}{\sqrt{2}l_s}\right). \quad \text{(S7)}$$

Function $\Phi(x)$ monotonously decreases with x. Thus, v_z decreases with increase of beam radius w_0 if one keeps power P_0 constant. It is also of interest to investigate the dependence of v_z on concentration of colloidal particles. For wide Gaussian beams ($w_0 > l_s$) the asymptotic expression for the function $\Phi(x)$ is $\Phi(x>>1)=\sqrt{\pi}/(2x)$. Taking into account that the second term in Eq.(S7) is $(w_0/a)^2$ times larger than the first one, the expression for the velocity of colloidal particles takes the form

$$v_z(\mathbf{r}\to 0)\approx\frac{1}{16}\sqrt{\frac{2}{\pi}}\frac{\sigma_{rp}}{\sigma_{ext}}\frac{P_0}{c\eta w_0},\quad w_0 > l_s . \quad \text{(S8)}$$

One can see from this expression that for wide beams the velocity of colloidal particles does not depend on their concentration. This statement may be not necessarily true for focused beams, but the previously made assumption that $a / f^{1/3} << w_0$ makes it impossible to ascertain this on the base of Eq.(S7).

2. Additional experiments on different colloidal system

To show the universality of advective transport, we performed experiments with different colloidal suspension. The suspension of 200 nm-diameter-silica particles (Geltech, Inc.) was made to reach the concentration of 1.5% v/v that corresponds to the average center to center interparticle separation of 4.4 particle's diameters. 4.5-μm-diameter polystyrene particles placed on the surface of the suspension were used for testing the transport abilities. The similar procedure as described in the main text was followed to deposit microspheres on the liquid interface. The prepared colloidal system was illuminated by s-polarized monochromatic (wavelength 532 nm, optical power 0.3W) unfocused beam at the incident angle of 27°. Upon the switch on of illumination, the target PS particles on the surface started instantaneously travel along the beam propagation direction (see Supplementary Video 2). The spatial distribution of the average velocity along the surface is shown in Fig. S1. As explained in the main text, this motion is mostly caused by the advective flow of colloidal particles. The maximum velocity of silica particles along the beam propagation direction measured in experiment is 16μm/s. The observed velocity in the case of silica colloid is slightly less than that of polystyrene (PS) ones (22.3μm/s). The lower velocity of silica particles (91μm/s for silica vs. 190μm/s for PS particles) is also found in numerical calculations.

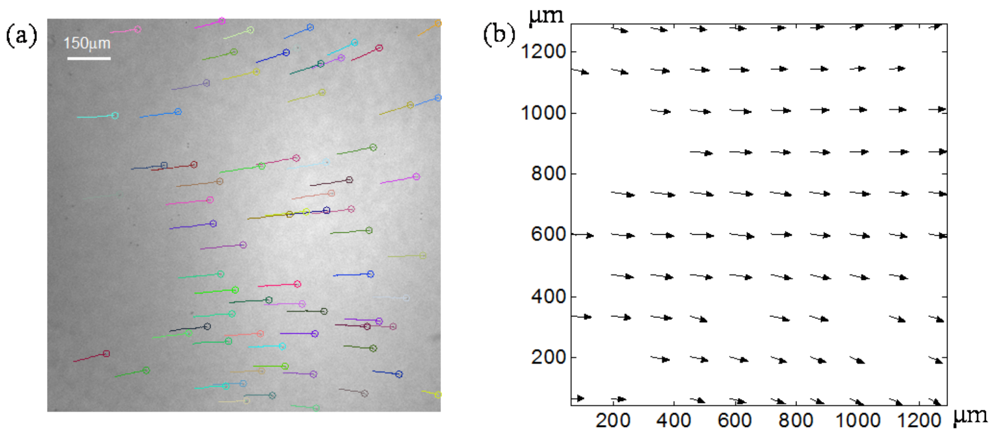

Figure S1 | Advective transport of target particles. (a) Trajectories of target particles during the 10s of laser illumination. The ends of trajectories are indicated by small circles. (b) Spatial distribution of time-averaged velocities of target PS particles on the surface of colloid. The length of the arrows is proportional to the speed magnitude while their orientations indicate the local direction of particles' motion.

3. Maximum achievable velocity

To estimate the maximum achievable velocity of advective flow, we performed extended numerical calculations with the same parameters as in experiment of the main text. As the volume fraction of particles used in the experiment is very low, the three- and higher multibody corrections are insignificant and we can use stokeslets approximation[4]. To take into account air-water interface, the mobility tensor in Eq. (S3) should be modified to take into account the presence of the surface. For a free interface, this can be done by introducing image particles[5, 6]:

$$\bar{\bar{\boldsymbol{\mu}}}_F(\mathbf{r}_i,\mathbf{r}_j) = \bar{\bar{\boldsymbol{\mu}}}(\mathbf{r}_i-\mathbf{r}_j) + \bar{\bar{\boldsymbol{\mu}}}(\mathbf{r}_i-\mathbf{r}'_j)\mathbf{R}_F\,. \quad \text{(S9)}$$

Here $\bar{\bar{\boldsymbol{\mu}}}_F(\mathbf{r}_i,\mathbf{r}_j)$ is the new mobility tensor, $\mathbf{R}_F = 1 - 2\hat{\mathbf{z}} \otimes \hat{\mathbf{z}}$ is the reflection operator with respect to water-air interface $z = 0$, $\mathbf{r}'_j = \mathbf{R}_F \mathbf{r}_j$ [5]. To find the distribution of coherent part of intensity I_{coh} in Eq.(S1), the colloid is regarded as an effective medium with a complex refractive index n_c where Re(n_c) is the refractive index of water and the imaginary part of refractive index $\mathrm{Im}(n_c) = 1/(2kl_s)$ depends on the scattering length and describes the attenuation of coherent wave into colloid. Thus, the refraction of a Gaussian beam into the colloidal system can be treated like in the case of an absorbing medium[7]. As the average interparticle separation of 700nm is much smaller than the characteristic dimensions of the Gaussian beam used in experiment ($w_0 = 1.1$ mm, $l_s = 0.4$ mm), the summation in Eq.(S3) can be replaced by integration. Using the Wolfram Mathematica software package, we estimated this integral numerically and typical results are illustrated in Figure S2 where the distribution of advective flows is shown both at the surface and in the bulk of the colloidal system.

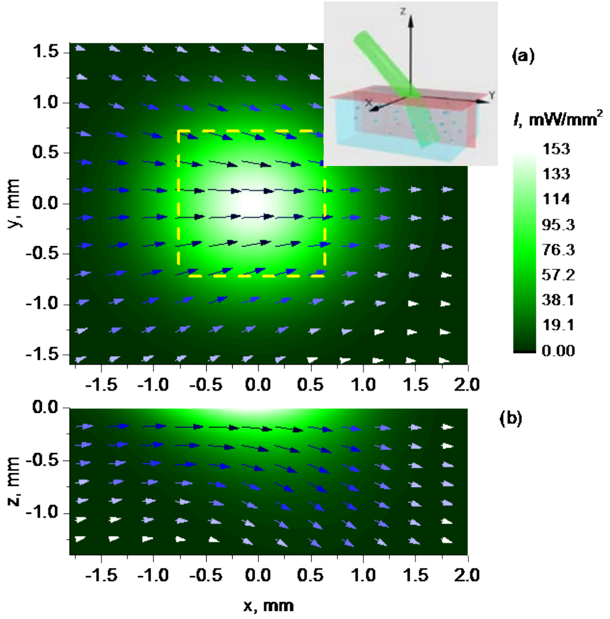

Figure S2 | Numerical calculation of advective flows. Distribution of intensity (color) and advective flow (arrows) along the surface (a) and perpendicular to the surface in a cross-section through the center of the beam (b). The length of the arrows is proportional to the local speed of the flow. The dashed rectangle in (a) shows the corresponding field of view in the experiment shown in Fig.1 of the main text. The insert shows position of cross-section planes.

As can be seen, the flow pattern is quite similar to the flow of target particles observed in the experiment. Moreover, the advective flows extend far beyond the range of action of optical forces demonstrating the effect of long-range hydrodynamic interactions. Calculations show that, because of these hydrodynamic interactions, the velocity of colloidal particles can achieve a maximum speed of the surface flow $\approx 190 \mu\mathrm{m/s}$ at the center of the beam.

4. Description of Supplementary Video 1

The video shows the motion of targeted 4.5μm PS particles on a surface of colloidal suspensions containing 0.3%v/v colloidal concentration of 200-nm-diameter polystyrene particles. The 0.3W s-polarized Gaussian laser beam with a diameter 2.2mm is incident at 27°. The motion of target particles is observed over an area of approximately 300 $\mu\mathrm{m} \times 200$ μm in the vicinity of the beam center located at the center of the frame.

5. Description of Supplementary Video 2

The video shows the motion of targeted 4.5μm PS particles on a surface of colloidal suspensions containing 1.5%v/v colloidal concentration of 200-nm-diameter silica particles. The 0.3W s-polarized Gaussian laser beam with a diameter 2.2mm is incident at 27°. The motion of target particles is observed over an area of approximately 300 μm×200 μm in the vicinity of the beam center located at the center of the frame.

References

1. Greenfield E, Nemirovsky J, El-Ganainy R, Christodoulides DN, Segev M. Shockwave based nonlinear optical manipulation in densely scattering opaque suspensions. *Optics Express* 2013, **21**(20)**:** 23785-23802.

2. Bohren CF, Huffman DR. *Absorption and scattering of light by small particles*. Willey-Interscience publication: NY, 1983.

3. Varadan V, Bringi V, Varadan V, Ishimaru A. Multiple scattering theory for waves in discrete random media and comparison with experiments. *Radio Science* 1983, **18**(3)**:** 321-327.

4. Happel J, Brenner H. *Low Reynolds number hydrodynamics: with special applications to particulate media*. Martinus Nijhoff: The Hague, 1983.

5. Cichocki B, Ekiel-Jeżewska ML, Wajnryb E. Hydrodynamic interactions between spheres in a viscous fluid with a flat free surface or hard wall. *The Journal of Chemical Physics* 2007, **126**(18)**:** 184704.

6. Perkins G, Jones R. Hydrodynamic interaction of a spherical particle with a planar boundary: II. Hard wall. *Physica A: Statistical Mechanics and its Applications* 1992, **189**(3)**:** 447-477.

7. Serdyuk VM, Titovitsky JA. A Simple Analytic Approximation for the Refracted Field at Gaussian Beam Incidence upon a Boundary of Absorbing Medium. *Journal of Electromagnetic Analysis and Applications* 2010, **2**(11)**:** 640-648.
